# Novel perspectives on the PHD-HIF oxygen sensing pathway in cardioprotection mediated by IPC and RIPC

**DOI:** 10.3389/fphys.2015.00137

**Published:** 2015-05-20

**Authors:** Silvia Martin-Puig, Daniel Tello, Julián Aragonés

**Affiliations:** ^1^Cell and Developmental Biology Department, Centro Nacional de Investigaciones CardiovascularesMadrid, Spain; ^2^Research Unit, Hospital Santa Cristina, Research Institute Princesa (IP), Autonomous University of MadridMadrid, Spain

**Keywords:** ischemic preconditioning, heart, PHD oxygen sensors, hypoxia-inducible factors, remote ischemic preconditioning

## Abstract

Reperfusion of ischemic cardiac tissue is the standard treatment for improving clinical outcome following myocardial infarction but is inevitably associated with ischemia-reperfusion injury (IRI). Ischemic myocardial injury can be alleviated by exposing the heart to brief episodes of sublethal ischemia-reperfusion prior to the ischemic insult, a phenomenon that has been termed ischemic preconditioning (IPC). Similarly, remote IPC (RIPC) is defined as transient episodes of ischemia at a distant site before a subsequent prolonged injury of the target organ. In this setting, adaptive responses to hypoxia/ischemia in peripheral tissues include the release of soluble factors that have the potential to protect cardiomyocytes remotely. Oxygen fluctuations is a hallmark of insufficient tissue perfusion and ischemic episodes. Emerging evidence indicates that prolyl hydroxylase oxygen sensors (PHDs) and hypoxia-inducible transcription factors (HIFs) are critical regulators of IPC and RIPC. In this review, we discuss recent findings concerning the role of the PHD-HIF axis in IPC and RIPC-mediated cardioprotection and examine molecular pathways and cell types that might be involved. We also appraise the therapeutic value of targeting the PHD-HIF axis to enhance cardiac tolerance against IRI.

## Ischemia-reperfusion injury

**Figure 1:**
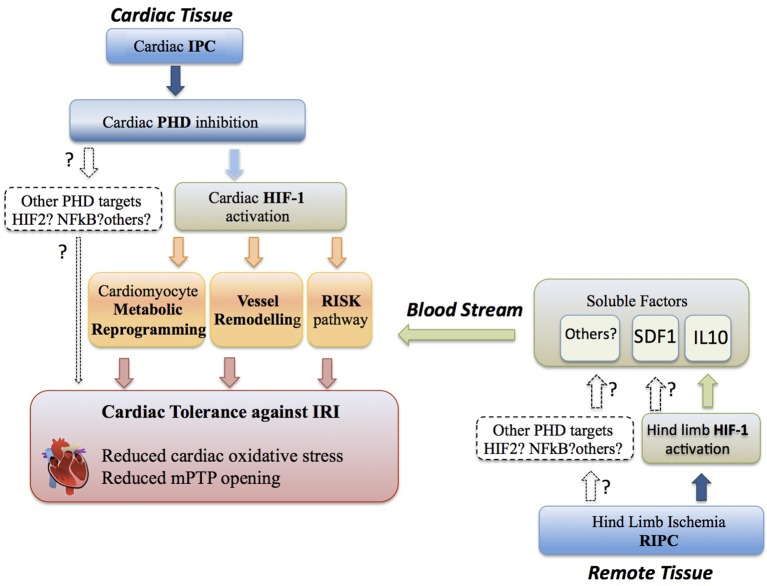
**Cardioprotection through the PHD-HIF pathway.** Cardiac tolerance against IRI (ischemia reperfusion injury) can be initiated by HIF1α activation locally in the cardiac tissue during IPC (ischemia preconditioning). IPC in the heart leads to cardiac PHD inhibition and subsequent induction of a HIF1α-dependent cardiac metabolic reprogramming, vessel remodeling and activation of the RISK pathway, which ultimately results in reduced cardiac oxidative stress, prevention of mitochondrial permeability transition pore (mPTP) opening and cardiac tissue salvage. The potential role of alternative PHD targets in IPC is indicated. Cardiac tolerance against IRI can be initiated by HIF1α activation at distance in non-cardiac tissue (i.e., hind limb) during RIPC (remote ischemia preconditioning). This remote HIF1α activation releases HIF-dependent soluble cardioprotectors (i.e., IL-10) from the remote tissue into the blood stream. These soluble factors initiate heart protection by activation of RISK pathways to confer cardiac tolerance against IRI, although additionally metabolic reprogramming and vessel remodeling could be potentially involved. The putative role of alternative PHD-dependent targets involved in RIPC is indicated.

Ischemic heart disease represents a heterogeneous group of pathological conditions characterized by insufficient perfusion of the heart. Coronary artery revascularization and reperfusion of ischemic cardiac tissue is the standard treatment in patients with myocardial infarction. However, reperfusion by itself causes additional tissue injury (termed ischemia-reperfusion injury, IRI) and is associated with a burst of reactive oxygen species (ROS) production and intracellular calcium overload (Garcia-Dorado et al., 1992; Kevin et al., 2003; Piper et al., 2006; Murphy and Steenbergen, 2008). Mitochondrial dysfunction plays a central role in cardiomyocyte damage during reperfusion injury (RI). The mitochondrial inner membrane is impermeable to most metabolites and ions; however, in certain stress conditions that can take place during IRI, such as increased calcium concentration, metabolic failure, or oxidative stress, the non-specific mitochondrial permeability transition pore (mPTP) opens in the inner mitochondrial membrane leading to cell death (Halestrap et al., 2004). IRI can be limited to some extent by subjecting the heart to short sublethal cycles of ischemia-reperfusion (ischemic preconditioning, IPC) prior to a harmful ischemic insult. Cardiac protection can also be induced by subjecting non-cardiac tissues (e.g., skeletal muscle) to a transient ischemic challenge (remote ischemic preconditioning, RIPC), resulting in the release of cardioprotective soluble factors by the remote ischemic tissue (Murry et al., 1986; Kharbanda et al., 2002). Given the relevance of oxygen fluctuations during sublethal and lethal ischemia-reperfusion cycles, the molecular pathways driven by the oxygen sensors prolyl hydroxylases (PHDs) and the hypoxia-inducible factors (HIFs) are extensively involved in the mechanisms underlying IPC (Figure 1). Moreover, emerging evidence suggests that activation of the PHD-HIF axis in RIPC leads to secretion of HIF-dependent cytoprotectors in peripheral tissues (e.g., skeletal muscle) that could ultimately protect the myocardium from IRI remotely (Figure 1). Here we review recent advances and perspectives on the role of these PHD-HIF oxygen-sensing pathways in cardioprotection during IPC and RIPC.

## The PHD-HIF oxygen-sensing pathway

The hypoxia-inducible transcription factors (HIF-1α, -2α, -3α) and the oxygen sensors (PHD 1, 2, 3, and factor inhibiting HIF, FIH) are key players in the cellular response to hypoxia. Insufficient oxygen supply to tissues rapidly increases the protein levels of HIFα subunits, which heterodimerize with their HIFß partner to activate transcription of a large number of target genes involved in the adaptive cellular and systemic response to oxygen deprivation (Semenza, 2007). HIFα subunits, however, do not sense oxygen directly. A family of oxygen-dependent 2-oxoglutarate dioxygenases that includes PHDs and factor inhibiting HIF (FIH) performs this activity. PHDs and FIH modulate HIF stabilization and transcriptional activation by hydroxylating specific prolyl or asparaginyl residues of HIFα subunits, respectively (Schofield and Ratcliffe, 2004). Under normal oxygen tensions, the hydroxylated prolyl residues of HIFα subunits serve as molecular marks for von Hippel-Lindau (VHL) protein, the recognition subunit of an E3 ubiquitin ligase complex, which labels HIFα subunits, through ubiquitination, for subsequent proteasomal degradation (Safran and Kaelin, 2003; Schofield and Ratcliffe, 2004). Under hypoxia, PHDs and FIH become inactive, resulting in stabilization of HIFα subunits. HIFα subunits then dimerizes with HIFß and translocates to the nucleus to recruit the transcriptional coactivator p300, which is essential for the transcriptional activity of HIFα (Lando et al., 2003). As numerous pathological conditions are associated with changes in oxygen tensions, there is a growing interest in understanding the role of the PHD-HIF axis in clinical settings, especially regarding its relevance in IPC- and RIPC-mediated cardioprotection.

## Cardiac PHD-HIF oxygen-sensing pathways in IPC

IPC is an effective strategy to protect the heart from IRI. This approach requires one or more short sublethal cycles of IR by coronary occlusion to protect the heart against a subsequent longer period of ischemia followed by reperfusion. Emerging evidence suggests that this phenomenon is mediated by the PHD-HIF oxygen-sensing pathway and has been reviewed elsewhere in detail (Fraisl et al., 2009). Thus, cardiac upregulation of HIF-1α occurs during IPC (Eckle et al., 2008), and partial deficiency or silencing of HIF-1α causes complete loss of IPC-induced protection against myocardial ischemia (Cai et al., 2008). Conversely, genetic inactivation or pharmacological inhibition of PHDs, leading to HIF-1α activation, confers cardiac tolerance to IRI (Eckle et al., 2007, 2008; Hyvarinen et al., 2010; Holscher et al., 2011; Ong et al., 2014b). Below, we discuss some of the HIF-dependent molecular responses that could be involved in this cardioprotective effect (Figure 1).

### Reprogramming of cardiac metabolism

Under conditions of limiting oxygen, cells reprogram their energy demands by promoting anaerobic metabolism. This response is mediated by HIF-dependent induction of glycolytic enzymes and also genes involved in the attenuation of oxidative metabolism such as pyruvate dehydrogenase kinases (PDK) -1, -3, and -4 (Semenza et al., 1994; Kim et al., 2006; Papandreou et al., 2006; Lu et al., 2008; Zhong et al., 2010), NDUFA4L2 (Tello et al., 2011), and the microRNA-210 (miR210) (Chan et al., 2009; Chen et al., 2010). Since reperfusion of ischemic tissues can lead to the formation of toxic ROS, triggering cell death, it has been proposed that HIF-dependent reduction of mitochondrial activity might help to counteract cell damage by attenuating the generation of mitochondrial ROS. Along this line, mice deficient for PHD1 present striking resistance to low flow hindlimb ischemia and liver IRI mimicking an IPC scenario (Aragones et al., 2008; Schneider et al., 2010). Protection seems to be associated with energetic reprogramming toward anaerobic metabolism, ultimately reducing mitochondrial oxygen consumption (Aragones et al., 2008; Schneider et al., 2010). Recent studies suggest that this anaerobic metabolic switch induced by PHDs could be relevant in cardiac IPC. Indeed, it has been observed that hearts from a PHD2 hypomorphic mouse (PHD2^gt/gt^), with partial reduction of PHD2, display a modest stabilization of HIF factors and a subsequent increase in some, but not all, metabolic genes previously identified as HIF-1α target genes (Hyvarinen et al., 2010). Accordingly, the expression of the glucose transporter-1 (Glut-1), phosphofructokinase-1 (Pfk-l), triosephosphate isomerase (Tpi), phosphoglycerate kinase-1 (Pgk-1) and Pdk-1 genes is specifically increased, indicating that these metabolic players could be particularly sensitive to modest elevations of HIF in PHD2^gt/gt^ heart. In line with this transcriptional program, pre-ischemic hearts of PHD2^gt/gt^ hypomorphic mice show enhanced lactate secretion as well as a mild mitochondrial swelling that does not compromise cardiac function. Importantly, when subjected to IR using an *ex vivo* Langendorff perfusion protocol, hearts from PHD2^gt/gt^ mice exhibit improved recovery of cardiac function and an increased amount of ATP (Hyvarinen et al., 2010), suggesting that their particular cardiac metabolic adaptation could be responsible for IRI tolerance. Moreover, additional studies in which PHD2 was either inactivated globally (Minamishima et al., 2008), or in a cardiomyocyte-restricted manner (Holscher et al., 2011), have also demonstrated submaximal HIF activation accompanied with a mild mitochondrial swelling (Minamishima et al., 2008) together with increased expression of Glut1, Pgk, Pfkl and Pdk1 genes and protection against myocardial infarction (Holscher et al., 2011). A recent study has addressed whether this metabolic reprogramming could be directly involved in HIF-dependent cardiac tolerance to IRI (Ong et al., 2014b). Accordingly, mice treated with the PHD inhibitor GSK360A have elevated cardiac expression of the glycolytic enzyme hexokinase II, which translocates to mitochondria to alleviate oxidative stress and inhibit mPTP opening (Ong et al., 2014b). Taken together, these findings suggest that an increased cardiac glycolytic rate upon heart activation of HIF could represent, by itself, a promising cardioprotector mechanism. Nonetheless, it is important to note that a more robust activation of HIF by vhl gene deletion (Lei et al., 2008) or simultaneous inactivation of PHD2 and PHD3 (Minamishima et al., 2008, 2009; Holscher et al., 2011) leads to a more marked repression of cardiac mitochondrial content, which although undoubtedly prevents the formation of deleterious mitochondrial ROS, also leads to the inevitable impairment of cardiac function (Lei et al., 2008; Minamishima et al., 2009). Moreover, chronic activation of the HIF-1α/PPARγ axis may contribute to cardiac hypertrophy and heart failure progression by promoting the switch from fatty acid utilization to glycolysis and subsequent lipid accumulation in the heart (Krishnan et al., 2009). Globally, these studies suggest that to confer cardioprotection, activation of the PHD-HIF pathway and subsequent metabolic reprogramming in the heart cannot exceed a certain level as disproportionate HIF-activation can lead to heart damage, cardiomegaly and impaired cardiac function (Lei et al., 2008; Minamishima et al., 2008, 2009; Holscher et al., 2011).

### Cardiac endothelial/vascular remodeling

Cardiomyocyte-specific PHD2-deficient mice present an increase in heart capillary density, which may positively contribute to cardioprotection after myocardial infarction (Holscher et al., 2011). Comparable findings are found in other mouse models associated with stronger cardiac HIF activation (Takeda et al., 2007; Lei et al., 2008; Minamishima et al., 2009; Holscher et al., 2011). Notwithstanding the intrinsic cardiac tolerance of PHD2^gt/gt^ mice to reperfusion described in the Langendorff model, endothelial cells have also been suggested to participate in the *in vivo* cardioprotection in these mice upon IRI (Hyvarinen et al., 2010). Expression of PHD2 mRNA in PHD2^gt/gt^ transgenic mice is reduced by 76% in cardiac endothelial cells, 77% in cardiac fibroblasts and almost 93% in cardiomyocytes, leading to cardiac stabilization of HIF-1α and HIF-2 α (Kerkela et al., 2013). These mice present an overall improved cardiac function measured as an increase in left ventricular (LV) ejection fraction after a 30-min cycle of ischemia by ligation of the left anterior descending (LAD) coronary artery followed by 24 h of reperfusion. Despite the similar capillary density in wild type and PHD2^gt/gt^ mice, the size of the coronary vessels is significantly enlarged and dilated in the transgenic mice, which might be associated with the significantly larger area of the LV perfused during ligation (Kerkela et al., 2013). Regarding the potential molecular mechanisms involved in the remodeling of vessels, inhibition of PHD2 in the heart correlates with increased expression of the endothelial genes angiopoietin-2 (Ang2), the angiopoietin receptor Tie-2, Apelin and Apelin receptor, and blockade of Tie2 signaling impairs vessel dilation and greatly abrogates cardiac protection after IRI. Nevertheless, in this study, the AR/LV (area at risk/left ventricle) ratio was not completely reversed in transgenic mice when Tie2 signaling was inhibited, resulting in increased vascular perfusion. Thus, additional vasodilator factors, such as nitric oxide (NO), may participate in the vascular phenotype and cardiac protection of these mice (Kerkela et al., 2013).

### The reperfusion injury signaling kinase (RISK) pathway

The RISK pathway comprises a group of pro-survival protein kinases (including Akt and Erk1/2) activated by receptor-dependent and-independent mechanisms, which attenuate mPTP opening and promote cardioprotection during the reperfusion stage of IR (Tsang et al., 2004; Park et al., 2006; Hausenloy and Yellon, 2007). Several studies have identified molecular links between HIF activity and the RISK pathway. Thus, PHD2-deficient hearts, or wild-type hearts subjected to IPC, upregulate key molecules of the adenosine signaling pathway, which are described to promote cardioprotection through the RISK pathway (Hausenloy and Yellon, 2007). Some adenosine signaling molecules include the A2B adenosine receptor (A2BAR) and the ecto-5′-nucleotidase CD73, which generates adenosine. Interestingly, CD73 expression is not activated in PHD2^gt/gt^ hypomorphic mice (Hyvarinen et al., 2010), suggesting that in this background cardioprotection might be obtained through adenosine-independent signaling, such as for instance the metabolic reprogramming mentioned above. Therefore, it would be pertinent to explore whether additional elements of the RISK pathway are activated in PHD2^gt/gt^ mice, or whether RISK activation requires a stronger cardiac HIF induction. An additional mediator of cardioprotection could be erythropoietin (Epo), the archetype HIF-responsive gene, which we recently found elevated in vhl-deficient hearts (Miro-Murillo et al., 2011). Indeed, several reports have shown that Epo protects cardiac tissue during ischemia and IR, particularly by overactivating the serine threonine kinase Akt, as well as through other pathways involving sonic hedgehog (Camici et al., 2007; Burger et al., 2009; Ueda et al., 2010; Ong et al., 2014a). Thus, it is conceivable that cardiac Epo expression could serve as a local autocrine or paracrine cardioprotector during IPC. In addition, several studies have also shown that cardiac HIF-1α expression can increase sublethal ROS production, resulting in oxidation/inactivation of the protein/lipid phosphatase and tensin homolog (PTEN), which negatively regulates the PI3K/Akt survival pathway (Cai and Semenza, 2005; Mocanu and Yellon, 2007; Cai et al., 2008). Therefore, HIF-1α could overactivate the prosurvival signaling cascades of Akt not only through extracellular soluble factors (e.g., adenosine and Epo), but also by ROS-mediated PTEN inactivation. The mechanisms by which Akt attenuates mPTP opening are still not completely understood, though recent studies suggest that Akt-dependent changes in mitochondrial morphology are required for Akt-dependent cardioprotection against IRI (Ong et al., 2014a,b).

### Nitric oxide signaling

Cardiac IPC upregulates the HIF-dependent gene inducible nitric oxide synthase (iNOS) and, importantly, iNOS deficiency abrogates the late phase of IPC (Guo et al., 1999). Increased intracellular nitric oxide (NO) as a consequence of endothelial/inducible NOS (eNOS)/iNOS activation plays a central role in cardiac IPC (Jones et al., 1999; Jung et al., 2000; Divisova et al., 2001; Vavrínková et al., 2001; Gao et al., 2002; Murphy and Steenbergen, 2008). At the molecular level, NO-dependent cardioprotection involves stimulation of guanylyl cyclase, which leads to cGMP production and subsequent activation of protein kinase G (PKG). Activated PKG in turn activates mitochondrial pKCε-dependent opening of mito-K_ATP_ channels (Costa et al., 2005), resulting in K^+^ influx, matrix alkalinization and subsequent ROS generation (Yao et al., 1999; Andrukhiv et al., 2006; Costa et al., 2008). Regarding the source of ROS, studies in isolated rat mitochondria have shown that intramitochondrial ROS generation is driven by complex I of the electron transport chain (Andrukhiv et al., 2006) and an earlier study using intact embryonic chick cardiomyocytes suggested the participation of complex III (Yao et al., 1999). Regardless of the mechanisms involved in their generation, mito-K_ATP_ channel-dependent sublethal ROS production is implicated in cardioprotection (Yao et al., 1999; Forbes et al., 2001; Gross and Lockwood, 2004; Shahid et al., 2008) because of their ability to activate an additional PKCε pool which ultimately inhibits mPTP opening (Costa et al., 2008). Thus, it is possible that PKG and iNOS constitute the molecular link between HIF and sublethal ROS-mediated PTEN inactivation and Akt activation. In fact, iNOS is phosphorylated and activated by Akt, and therefore the iNOS-NO-PKG molecular axis has been positioned as a downstream effector of Akt in IPC (Hausenloy et al., 2005). Also, local cardiac NO release may influence vascular tone and thus impact cardiac perfusion upon IRI (Fraisl, 2013).

## PHD-HIF oxygen sensing pathways in RIPC

### HIF-dependent soluble factors in RIPC

In addition to the phenomenon of IPC described earlier, recent observations indicate that transient and local exposure to hypoxia in certain tissues (limb skeletal muscle, brain, intestine, and kidney) are protective against future ischemic insults to organs and tissues distant from the regional area of initial ischemia, with similar efficacy as local IPC. This unique and innovative treatment approach has been named remote ischemic preconditioning or RIPC (Bolte et al., 2007; Randhawa and Jaggi, 2014). The potential clinical applications of this endogenous adaptive capacity are enormous and are especially attractive in the setting of clinical interventions to resolve highly prevalent cardiovascular diseases such as coronary obstruction, heart failure or stroke, where tissue reperfusion after an ischemic insult could have devastating consequences (Bolte et al., 2007; Randhawa and Jaggi, 2014). As a simple, inexpensive and relatively uncomplicated approach to reduce cardiovascular perioperative ischemic damage, there is a growing interest in understanding the molecular mediators (i.e., soluble factors) involved in ischemic protection to improve and develop novel therapeutic strategies. Results from preclinical studies have shown that transient occlusion of cerebral, mesenteric or intestinal arteries, as well as the abdominal aorta or skeletal muscle, promotes cardioprotection against IRI by myocardial preconditioning (Randhawa and Jaggi, 2014). In spite of the clinical benefits reported to the date, identification of the protective factors involved in RIPC and how they impact on myocardial survival upon reperfusion remains largely unknown; nevertheless, some novel soluble factors have emerged in the setting of RIPC and recent studies point to a role for the PHD-HIF pathway in RIPC (Figure 1). Stabilization of HIF-1α protein in the heart after remote ischemic injury has been confirmed both in basic models (Cai et al., 2013; Kalakech et al., 2013) and also in human clinical trials (Albrecht et al., 2013; Randhawa and Jaggi, 2014). The possibility that HIF-1 could control the expression of soluble factors to promote cardioprotection in RIPC was initially tested in muscle tissue using gene delivery of HIF-1 (Czibik et al., 2009). Additionally, a positive role for HIF-1 was suggested by experiments showing that HIF-1α-deficient mice completely lose RIPC-induced cardioprotection after transient ligation of the femoral artery (Cai et al., 2013). Using a similar experimental approach based on heterozygous HIF-1α heterozygous mice and pharmacological inhibition of HIF with cadmium, Kalakech and colleagues reported that HIF-1 was unnecessary in acute RIPC (Kalakech et al., 2013). A potential explanation for these discrepancies could be the subtle technical differences in the hindlimb ischemia protocol to induce RIPC, infarct size determination or the time between the transient ligation of the femoral artery and coronary occlusion. Moreover, despite the dramatic reduction of HIF-1α protein in the limbs of HIF-1α heterozygous mice after RIPC or cadmium treatment, HIF-1α levels were above the control condition. Because all or part of the effects mediated by the secreted component of the RIPC response may be dose dependent, it cannot be ruled out that this minimum amount of HIF-1 may be enough to activate the paracrine-protective mechanism in response to myocardial infarction. Regarding the HIF-dependent soluble factors that could mediate RIPC, Cai and colleagues showed that interleukin-10 (IL-10) is a primary HIF-1α target in murine cardiomyocytes subjected to cyclic hypoxia-reoxygenation. Furthermore, transient femoral ligation increases IL-10 plasma concentration and promotes a subsequent remote activation of cardiac Akt signaling. This effect is inhibited in HIF-1α heterozygous deficient mice, suggesting that IL-10 could be responsible for the activation of Akt pathway in the distant heart and subsequent cardioprotection (Cai et al., 2013). In line with the characterization of mechanisms mediating cardioprotection by IL-10, it has been reported that this cytokine can prevent hypertrophic remodeling and improve cardiac function in a model of isoproterenol-induced pressure overload by controling Stat-3 activation and promoting NFκ B inhibition (Verma et al., 2012). In addition, the group of Pawan K. Singal has shown that IL-10 promotes Stat-3 activation associated with Akt phosphorylation in cardiomyocytes (Dhingra et al., 2011). Furthermore, when IL-10-stimulated cardiomyocytes are treated with the ERK1/2 MAPK inhibitor PD 98059, the cardioprotection mediated by this anti-inflammatory cytokine is lost (Dhingra et al., 2009). Also, a recent study has shown that IL-10 activates Toll-like receptor 4 and mediates cardiomyocyte survival in a MyD88-interferon regulatory factor 3 (IRF3)-NFκB dependent manner (Bagchi et al., 2013). However, it is important to consider the potential roles of additional HIF-dependent genes in RIPC. Accordingly, hindlimb ischemia also elevates in plasma the stromal cell-derived factor 1 (SDF-1) also known as C-X-C motif chemokine 12 (CXCL12), which mediates cardioprotection through its receptor CXCR4 (Davidson et al., 2013). Indeed, this study demonstrated that AMD3100, a highly specific inhibitor of CXCR4, blocked RIPC upon transient femoral ligation, suggesting that IL-10 and SDF1 could act in concert to confer HIF-dependent cardioprotection at a distance.

Finally, cardioprotection after RIPC could be mediated by additional HIF-1α–independent signaling involving soluble factors, such as HIF-2α stabilization and subsequent Epo production (Kapitsinou et al., 2014) or NFκB signaling (Kant et al., 2008). Along this line, one of the original signals described in IRI models, mito-K_ATP_ channel opening, was found to be a downstream mediator of NFκB activation in a rat model of renal RIPC, with positive effects for cardioprotection (Diwan et al., 2008). In terms of pharmacological HIF-PHD inhibition strategies in the setting of RIPC, it is important to appreciate that NFκB signaling is targeted and activated upon PHD inhibition (Cummins et al., 2006), potentially contributing to the secretion of inflammatory mediators that might be critical in the evolution of the ischemic insult upon RIPC. Nonetheless, the relative contribution of PHD-dependent but HIF-1-independent pathways in cardiac protection remains poorly explored and warrants further investigation.

### Cardiac cell types potentially involved in RIPC

In addition to cardiomycoytes, the heart is a complex structure composed of many cell types. In the setting of RIPC initiated by extracellular soluble factors, aside from cardiomyocytes, other heart cell types might also respond to cytoprotectors to mediate global cardiac protection. As discussed earlier, the vascular endothelium is an essential element mediating IPC salvage and, in the setting of RIPC, it might also be prudent to determine if some or any of the described cardiac secreted factors (IL-10, SDF1, Epo, etc.) could directly impact endothelial cells to mediate heart protection by regulating the coronary diameter, endothelial permeability or vascular tone. In this respect, the presence of Epo receptors on endothelial cells and the activation of the PI3K/Akt pathway in response to Epo have been previously evaluated (Marzo et al., 2008). Additionally, IL-10 has been described to protect against aging or inflammation-mediated endothelial dysfunction (Kinzenbaw et al., 2013) and SDF1 has been reported to increase capillary density in a murine model of myocardial infarction (Ziegler et al., 2012). Another heart component potentially mediating cardioprotection upon RIPC is the epicardium, the outer most layer of the heart that, during development, contributes to the formation of the coronary vasculature and interstitial cardiac fibroblasts (Martin-Puig et al., 2008). Interestingly, upon myocardial infarction, murine epicardial-derived cells (EPDCs) secrete a plethora of paracrine factors mostly related to angiogenesis (Vegfa, Angpt1, Ang, Fgf1, Fgf2, Fgf9, Pdgfa, Pdgfc, Pdgf) among others (Adamts1, Sdf1, Mcp1, and IL-6) and are able to proliferate and migrate toward the infarcted myocardium, giving rise to perivascular fibroblasts and smooth muscle cells (Zhou et al., 2011). Remarkably, this paracrine response is associated with a reduction of infarct size and improvement in cardiac function (Zhou et al., 2011). Since some of these secreted molecules are HIF targets and others, for instance SDF1, have previously been described as beneficial factors produced upon RIPC, EPDCs could reasonably be mediating part of the cardiac protection provided by RIPC.

Finally, the involvement of soluble mediators such as active cytokines in the cardioprotective effect of RIPC, points to the potential modulation of different aspects of the immune system that could contribute to their beneficial effects. Thus, it will be of special interest to identify the cytokine signature upon RIPC that could modulate immune cell phenotypes such as M1/M2 macrophages and Th17/Treg lymphocyte polarization. Indeed, the participation of NFκB signaling in RIPC cardioprotection (Diwan et al., 2008; Kant et al., 2008) offers a potential interplay between RIPC, the PHD-HIF pathway and modulation of the immune system.

## Concluding remarks and therapeutic opportunities

Emerging evidence suggests that the PHD-HIF oxygen-sensing pathway induces cardiac intracellular responses (metabolic reprogramming) as well as systemic protective mechanisms initiated by extracellular factors (adenosine, Epo, NO, etc.), which contribute to myocardium salvage during reperfusion. The extracellular component of cardioprotection is strongly supported by the benefits of RIPC that rely on soluble factors released by peripheral ischemic tissues (e.g., IL-10 and SDF1), which subsequently activate cardioprotective RISK pathways in cardiac tissue (Figure 1). Thus, in the setting of IPC or RIPC, it might be important firstly to identify novel HIF-dependent soluble cardioprotectors and, secondly, to investigate the impact of these extracellular factors on the coronary endothelium regulating tissue perfusion, or through additional mechanisms, to promote cardioprotection. Importantly, pharmacological inhibition or genetic inactivation of PHD oxygen sensors has been shown to confer ischemic tolerance in heart tissue, which could have clinical impact in countering IR damage. Notwithstanding the potential cardioprotection offered by global HIF activation after systemic PHD pharmacological inhibition, some deleterious side effects may occur when cardiac HIFs are overactivated such as reduced cardiac mitochondrial function or lipid accumulation in the heart. An alternative therapeutic opportunity might be focused on local chemical inhibition of PHDs in remote tissues (e.g., skeletal muscle) in an attempt to mimic the endogenous RIPC response. A caveat to these approaches, however, is that a HIF-dependent cardioprotective program requires some time (perhaps hours), which could limit to some extent its clinical value when the ischemia/reperfusion episode is taking place. A more specific approach could exploit HIF-dependent soluble/secreted factors involved in IPC (adenosine) or RIPC (IL-10, SDF1) as therapies. Indeed, alone or in combination, these factors could be administered in the reperfused cardiac tissue when the occluded coronary artery is reopened. Along this line, the administration of some HIF-dependent soluble factors, such as adenosine or Epo, have been clinically tested in humans although the clinical efficacy has not been fully addressed (Singh et al., 2012; Mastromarino et al., 2013; Talan and Latini, 2013; Garcia-Dorado et al., 2014; Lund et al., 2014) and further research needs to be conducted in terms of posology and time of administation. A better understanding of the molecular mechanisms linking HIF-secreted factors to cardioprotection and the identification of novel HIF-dependent extracellular molecules will help to overcome these limitations and improve clinical benefits toward salvage of ischemic myocardium.

### Conflict of interest statement

The authors declare that the research was conducted in the absence of any commercial or financial relationships that could be construed as a potential conflict of interest.

## References

[B1] Albrecht M. Zitta K. Bein B. Wennemuth G. Broch O. Renner J.. (2013). Remote ischemic preconditioning regulates HIF-1alpha levels, apoptosis and inflammation in heart tissue of cardiosurgical patients: a pilot experimental study. Basic Res. Cardiol. 108, 314. 10.1007/s00395-012-0314-0 23203207

[B80] Andrukhiv A. Costa A. D. West I. C. Garlid K. D. (2006). Opening mitoKATP increases superoxide generation from complex I of the electron transport chain. Am. J. Physiol. Heart. Circ. Physiol. 291, H2067–H2074. 10.1152/ajpheart.00272.2006 16798828

[B2] Aragones J. Schneider M. Van Geyte K. Fraisl P. Dresselaers T. Mazzone M.. (2008). Deficiency or inhibition of oxygen sensor Phd1 induces hypoxia tolerance by reprogramming basal metabolism. Nat. Genet. 40, 170–180. 10.1038/ng.2007.62 18176562

[B3] Bagchi A. K. Sharma A. Dhingra S. Lehenbauer Ludke A. R. Al-Shudiefat A. A. Singal P. K. (2013). Interleukin-10 activates Toll-like receptor 4 and requires MyD88 for cardiomyocyte survival. Cytokine 61, 304–314. 10.1016/j.cyto.2012.10.013 23141143

[B4] Bolte C. S. Liao S. Gross G. J. Schultz Jel J. (2007). Remote preconditioning-endocrine factors in organ protection against ischemic injury. Endocr. Metab. Immune Disord. Drug Targets 7, 167–175. 10.2174/187153007781662585 17897043

[B5] Burger D. Xenocostas A. Feng Q. P. (2009). Molecular basis of cardioprotection by erythropoietin. Curr. Mol. Pharmacol. 2, 56–69. 10.2174/1874467210902010056 20021446

[B6] Cai Z. Luo W. Zhan H. Semenza G. L. (2013). Hypoxia-inducible factor 1 is required for remote ischemic preconditioning of the heart. Proc. Natl. Acad. Sci. U.S.A. 110, 17462–17467. 10.1073/pnas.1317158110 24101519 PMC3808664

[B7] Cai Z. Semenza G. L. (2005). PTEN activity is modulated during ischemia and reperfusion: involvement in the induction and decay of preconditioning. Circ. Res. 97, 1351–1359. 10.1161/01.RES.0000195656.52760.30 16284183

[B8] Cai Z. Zhong H. Bosch-Marce M. Fox-Talbot K. Wang L. Wei C.. (2008). Complete loss of ischaemic preconditioning-induced cardioprotection in mice with partial deficiency of HIF-1 alpha. Cardiovasc. Res. 77, 463–470. 10.1093/cvr/cvm035 18006459

[B9] Camici G. G. Stallmach T. Hermann M. Hassink R. Doevendans P. Grenacher B.. (2007). Constitutively overexpressed erythropoietin reduces infarct size in a mouse model of permanent coronary artery ligation. Methods Enzymol. 435, 147–155. 10.1016/S0076-6879(07)35008-8 17998053

[B10] Chan S. Y. Zhang Y. Y. Hemann C. Mahoney C. E. Zweier J. L. Loscalzo J. (2009). MicroRNA-210 controls mitochondrial metabolism during hypoxia by repressing the iron-sulfur cluster assembly proteins ISCU1/2. Cell Metab. 10, 273–284. 10.1016/j.cmet.2009.08.015 19808020 PMC2759401

[B11] Chen Z. Li Y. Zhang H. Huang P. Luthra R. (2010). Hypoxia-regulated microRNA-210 modulates mitochondrial function and decreases ISCU and COX10 expression. Oncogene 29, 4362–4368. 10.1038/onc.2010.193 20498629

[B12] Costa A. D. Garlid K. D. West I. C. Lincoln T. M. Downey J. M. Cohen M. V.. (2005). Protein kinase G transmits the cardioprotective signal from cytosol to mitochondria. Circ. Res. 97, 329–336. 10.1161/01.RES.0000178451.08719.5b 16037573

[B78] Costa A. D. Pierre S. V. Cohen M. V. Downey J. M. Garlid K. D. (2008). cGMP signalling in pre- and post-conditioning: the role of mitochondria. Cardiovasc Res. 77, 344–352. 10.1093/cvr/cvm050 18006449

[B13] Cummins E. P. Berra E. Comerford K. M. Ginouves A. Fitzgerald K. T. Seeballuck F.. (2006). Prolyl hydroxylase-1 negatively regulates IkappaB kinase-beta, giving insight into hypoxia-induced NFkappaB activity. Proc. Natl. Acad. Sci. U.S.A. 103, 18154–18159. 10.1073/pnas.0602235103 17114296 PMC1643842

[B14] Czibik G. Martinov V. Ruusalepp A. Sagave J. Skare O. Valen G. (2009). *In vivo* remote delivery of DNA encoding for hypoxia-inducible factor 1 alpha reduces myocardial infarct size. Clin. Transl. Sci. 2, 33–40. 10.1111/j.1752-8062.2008.00077.x 20443865 PMC5350792

[B15] Davidson S. M. Selvaraj P. He D. Boi-Doku C. Yellon R. L. Vicencio J. M.. (2013). Remote ischaemic preconditioning involves signalling through the SDF-1alpha/CXCR4 signalling axis. Basic Res. Cardiol. 108, 377. 10.1007/s00395-013-0377-6 23917520

[B16] Dhingra S. Bagchi A. K. Ludke A. L. Sharma A. K. Singal P. K. (2011). Akt regulates IL-10 mediated suppression of TNFalpha-induced cardiomyocyte apoptosis by upregulating Stat3 phosphorylation. PLoS ONE 6:e25009. 10.1371/journal.pone.0025009 21949832 PMC3176791

[B17] Dhingra S. Sharma A. K. Arora R. C. Slezak J. Singal P. K. (2009). IL-10 attenuates TNF-alpha-induced NF kappaB pathway activation and cardiomyocyte apoptosis. Cardiovasc. Res. 82, 59–66. 10.1093/cvr/cvp040 19181934

[B18] Divisova J. Vavrinkova H. Tutterova M. Kazdova L. Meschisvili E. (2001). Effect of ACE inhibitor captopril and L-arginine on the metabolism and on ischemia-reperfusion injury of the isolated rat heart. Physiol. Res. 50, 143–152. 11522042

[B19] Diwan V. Kant R. Jaggi A. S. Singh N. Singh D. (2008). Signal mechanism activated by erythropoietin preconditioning and remote renal preconditioning-induced cardioprotection. Mol. Cell. Biochem. 315, 195–201. 10.1007/s11010-008-9808-3 18528635

[B20] Eckle T. Kohler D. Lehmann R. El Kasmi K. Eltzschig H. K. (2008). Hypoxia-inducible factor-1 is central to cardioprotection: a new paradigm for ischemic preconditioning. Circulation 118, 166–175. 10.1161/CIRCULATIONAHA.107.758516 18591435

[B21] Eckle T. Krahn T. Grenz A. Kohler D. Mittelbronn M. Ledent C.. (2007). Cardioprotection by ecto-5′-nucleotidase (CD73) and A2B adenosine receptors. Circulation 115, 1581–1590. 10.1161/CIRCULATIONAHA.106.669697 17353435

[B82] Forbes R. A. Steenbergen C. Murphy E. (2001). Diazoxide-induced cardioprotection requires signaling through a redox-sensitive mechanism. Circ. Res. 88, 802–809. 11325872 10.1161/hh0801.089342

[B22] Fraisl P. (2013). Crosstalk between oxygen- and nitric oxide-dependent signaling pathways in angiogenesis. Exp. Cell Res. 319, 1331–1339. 10.1016/j.yexcr.2013.02.010 23485765

[B23] Fraisl P. Aragones J. Carmeliet P. (2009). Inhibition of oxygen sensors as a therapeutic strategy for ischaemic and inflammatory disease. Nat. Rev. Drug Discov. 8, 139–152. 10.1038/nrd2761 19165233

[B24] Gao F. Gao E. Yue T. L. Ohlstein E. H. Lopez B. L. Christopher T. A.. (2002). Nitric oxide mediates the antiapoptotic effect of insulin in myocardial ischemia-reperfusion: the roles of PI3-kinase, Akt, and endothelial nitric oxide synthase phosphorylation. Circulation 105, 1497–1502. 10.1161/01.CIR.0000012529.00367.0F 11914261

[B25] Garcia-Dorado D. Garcia-del-Blanco B. Otaegui I. Rodriguez-Palomares J. Pineda V. Gimeno F.. (2014). Intracoronary injection of adenosine before reperfusion in patients with ST-segment elevation myocardial infarction: a randomized controlled clinical trial. Int. J. Cardiol. 177, 935–941. 10.1016/j.ijcard.2014.09.203 25449504

[B26] Garcia-Dorado D. Theroux P. Duran J. M. Solares J. Alonso J. Sanz E.. (1992). Selective inhibition of the contractile apparatus. A new approach to modification of infarct size, infarct composition, and infarct geometry during coronary artery occlusion and reperfusion. Circulation 85, 1160–1174. 10.1161/01.CIR.85.3.1160 1537114

[B83] Gross G. J. Lockwood S. F. (2004). Cardioprotection and myocardial salvage by a disodium disuccinate astaxanthin derivative (Cardax). Life Sci. 75, 215–224. 10.1016/j.lfs.2003.12.006 15120573

[B27] Guo Y. Jones W. K. Xuan Y. T. Tang X. L. Bao W. Wu W. J.. (1999). The late phase of ischemic preconditioning is abrogated by targeted disruption of the inducible NO synthase gene. Proc. Natl. Acad. Sci. U.S.A. 96, 11507–11512. 10.1073/pnas.96.20.11507 10500207 PMC18064

[B28] Halestrap A. P. Clarke S. J. Javadov S. A. (2004). Mitochondrial permeability transition pore opening during myocardial reperfusion–a target for cardioprotection. Cardiovasc. Res. 61, 372–385. 10.1016/S0008-6363(03)00533-9 14962470

[B29] Hausenloy D. J. Tsang A. Mocanu M. M. Yellon D. M. (2005). Ischemic preconditioning protects by activating prosurvival kinases at reperfusion. Am. J. Physiol. Heart Circ. Physiol. 288, H971–H976. 10.1152/ajpheart.00374.2004 15358610

[B30] Hausenloy D. J. Yellon D. M. (2007). Reperfusion injury salvage kinase signalling: taking a RISK for cardioprotection. Heart Fail. Rev. 12, 217–234. 10.1007/s10741-007-9026-1 17541822

[B31] Holscher M. Silter M. Krull S. von Ahlen M. Hesse A. Schwartz P.. (2011). Cardiomyocyte-specific prolyl-4-hydroxylase domain 2 knock out protects from acute myocardial ischemic injury. J. Biol. Chem. 286, 11185–11194. 10.1074/jbc.M110.186809 21270129 PMC3064173

[B32] Hyvarinen J. Hassinen I. E. Sormunen R. Maki J. M. Kivirikko K. I. Koivunen P.. (2010). Hearts of hypoxia-inducible factor prolyl 4-hydroxylase-2 hypomorphic mice show protection against acute ischemia-reperfusion injury. J. Biol. Chem. 285, 13646–13657. 10.1074/jbc.M109.084855 20185832 PMC2859527

[B33] Jones S. P. Girod W. G. Palazzo A. J. Granger D. N. Grisham M. B. Jourd'Heuil D.. (1999). Myocardial ischemia-reperfusion injury is exacerbated in absence of endothelial cell nitric oxide synthase. Am. J. Physiol. 276(5 Pt 2), H1567–1573. 10330240 10.1152/ajpheart.1999.276.5.H1567

[B34] Jung F. Palmer L. A. Zhou N. Johns R. A. (2000). Hypoxic regulation of inducible nitric oxide synthase via hypoxia inducible factor-1 in cardiac myocytes. Circ. Res. 86, 319–325. 10.1161/01.RES.86.3.319 10679484

[B35] Kalakech H. Tamareille S. Pons S. Godin-Ribuot D. Carmeliet P. Furber A.. (2013). Role of hypoxia inducible factor-1alpha in remote limb ischemic preconditioning. J. Mol. Cell. Cardiol. 65, 98–104. 10.1016/j.yjmcc.2013.10.001 24140799

[B36] Kant R. Diwan V. Jaggi A. S. Singh N. Singh D. (2008). Remote renal preconditioning-induced cardioprotection: a key role of hypoxia inducible factor-prolyl 4-hydroxylases. Mol. Cell. Biochem. 312, 25–31. 10.1007/s11010-008-9717-5 18273560

[B37] Kapitsinou P. P. Sano H. Michael M. Kobayashi H. Davidoff O. Bian A.. (2014). Endothelial HIF-2 mediates protection and recovery from ischemic kidney injury. J. Clin. Invest. 124, 2396–2409. 10.1172/JCI69073 24789906 PMC4092875

[B38] Kerkela R. Karsikas S. Szabo Z. Serpi R. Magga J. Gao E.. (2013). Activation of hypoxia response in endothelial cells contributes to ischemic cardioprotection. Mol. Cell. Biol. 33, 3321–3329. 10.1128/MCB.00432-13 23775121 PMC3753900

[B39] Kevin L. G. Camara A. K. Riess M. L. Novalija E. Stowe D. F. (2003). Ischemic preconditioning alters real-time measure of O2 radicals in intact hearts with ischemia and reperfusion. Am. J. Physiol. Heart Circ. Physiol. 284, H566–H574. 10.1152/ajpheart.00711.2002 12414448

[B40] Kharbanda R. K. Mortensen U. M. White P. A. Kristiansen S. B. Schmidt M. R. Hoschtitzky J. A.. (2002). Transient limb ischemia induces remote ischemic preconditioning *in vivo*. Circulation 106, 2881–2883. 10.1161/01.CIR.0000043806.51912.9B 12460865

[B87] Kim J. W. Tchernyshyov I. Semenza G. L. Dang C. V. (2006). HIF-1-mediated expression of pyruvate dehydrogenase kinase: a metabolic switch required for cellular adaptation to hypoxia. Cell Metab. 3, 177–185. 10.1016/j.cmet.2006.02.002 16517405

[B41] Kinzenbaw D. A. Chu Y. Pena Silva R. A. Didion S. P. Faraci F. M. (2013). Interleukin-10 protects against aging-induced endothelial dysfunction. Physiol. Rep. 1, e00149. 10.1002/phy2.149 24400151 PMC3871464

[B42] Krishnan J. Suter M. Windak R. Krebs T. Felley A. Montessuit C.. (2009). Activation of a HIF1alpha-PPARgamma axis underlies the integration of glycolytic and lipid anabolic pathways in pathologic cardiac hypertrophy. Cell Metab. 9, 512–524. 10.1016/j.cmet.2009.05.005 19490906

[B43] Lando D. Goman J. J. Whitelaw M. L. Peet D. J. (2003). Oxygen-dependent regulation of hypoxia-inducible factors by prolyl and asparaginyl hydroxylation. Eur. J. Biochem. 270, 781–790. 10.1046/j.1432-1033.2003.03445.x 12603311

[B44] Lei L. Mason S. Liu D. Huang Y. Marks C. Hickey R.. (2008). Hypoxia-inducible factor-dependent degeneration, failure, and malignant transformation of the heart in the absence of the von Hippel-Lindau protein. Mol. Cell. Biol. 28, 3790–3803. 10.1128/MCB.01580-07 18285456 PMC2423296

[B45] Lu C. W. Lin S. C. Chen K. F. Lai Y. Y. Tsai S. J. (2008). Induction of pyruvate dehydrogenase kinase-3 by hypoxia-inducible factor-1 promotes metabolic switch and drug resistance. J. Biol. Chem. 283, 28106–28114. 10.1074/jbc.M803508200 18718909 PMC2661383

[B46] Lund A. Lundby C. Olsen N. V. (2014). High-dose erythropoietin for tissue protection. Eur. J. Clin. Invest. 44, 1230–1238. 10.1111/eci.12357 25345962

[B47] Martin-Puig S. Wang Z. Chien K. R. (2008). Lives of a heart cell: tracing the origins of cardiac progenitors. Cell Stem Cell 2, 320–331. 10.1016/j.stem.2008.03.010 18397752

[B48] Marzo F. Lavorgna A. Coluzzi G. Santucci E. Tarantino F. Rio T.. (2008). Erythropoietin in heart and vessels: focus on transcription and signalling pathways. J. Thromb. Thrombolysis 26, 183–187. 10.1007/s11239-008-0212-3 18338108

[B49] Mastromarino V. Musumeci M. B. Conti E. Tocci G. Volpe M. (2013). Erythropoietin in cardiac disease: effective or harmful? J. Cardiovasc. Med. (Hagerstown) 14, 870–878. 10.2459/JCM.0b013e328362c6ae 23811836

[B50] Minamishima Y. A. Moslehi J. Bardeesy N. Cullen D. Bronson R. T. Kaelin W. G. Jr. (2008). Somatic inactivation of the PHD2 prolyl hydroxylase causes polycythemia and congestive heart failure. Blood 111, 3236–3244. 10.1182/blood-2007-10-117812 18096761 PMC2265460

[B51] Minamishima Y. A. Moslehi J. Padera R. F. Bronson R. T. Liao R. Kaelin W. G. Jr. (2009). A feedback loop involving the Phd3 prolyl hydroxylase tunes the mammalian hypoxic response *in vivo*. Mol. Cell. Biol. 29, 5729–5741. 10.1128/MCB.00331-09 19720742 PMC2772748

[B52] Miro-Murillo M. Elorza A. Soro-Arnaiz I. Albacete-Albacete L. Ordonez A. Balsa E.. (2011). Acute Vhl gene inactivation induces cardiac HIF-dependent erythropoietin gene expression. PLoS ONE 6:e22589. 10.1371/journal.pone.0022589 21811636 PMC3141062

[B53] Mocanu M. M. Yellon D. M. (2007). PTEN, the Achilles' heel of myocardial ischaemia/reperfusion injury? Br. J. Pharmacol. 150, 833–838. 10.1038/sj.bjp.0707155 17293884 PMC2013879

[B54] Murphy E. Steenbergen C. (2008). Mechanisms underlying acute protection from cardiac ischemia-reperfusion injury. Physiol. Rev. 88, 581–609. 10.1152/physrev.00024.2007 18391174 PMC3199571

[B55] Murry C. E. Jennings R. B. Reimer K. A. (1986). Preconditioning with ischemia: a delay of lethal cell injury in ischemic myocardium. Circulation 74, 1124–1136. 10.1161/01.CIR.74.5.1124 3769170

[B56] Ong S. B. Hall A. R. Dongworth R. K. Kalkhoran S. Pyakurel A. Scorrano L.. (2014a). Akt protects the heart against ischaemia-reperfusion injury by modulating mitochondrial morphology. Thromb. Haemost. 113, 513–521. 10.1160/TH14-07-0592 25253080

[B57] Ong S. G. Lee W. H. Theodorou L. Kodo K. Lim S. Y. Shukla D. H.. (2014b). HIF-1 reduces ischaemia-reperfusion injury in the heart by targeting the mitochondrial permeability transition pore. Cardiovasc. Res. 104, 24–36. 10.1093/cvr/cvu172 25063991

[B58] Papandreou I. Cairns R. A. Fontana L. Lim A. L. Denko N. C. (2006). HIF-1 mediates adaptation to hypoxia by actively downregulating mitochondrial oxygen consumption. Cell Metab. 3, 187–197. 10.1016/j.cmet.2006.01.012 16517406

[B59] Park S. S. Zhao H. Mueller R. A. Xu Z. (2006). Bradykinin prevents reperfusion injury by targeting mitochondrial permeability transition pore through glycogen synthase kinase 3beta. J. Mol. Cell. Cardiol. 40, 708–716. 10.1016/j.yjmcc.2006.01.024 16516918

[B60] Piper H. M. Kasseckert S. Abdallah Y. (2006). The sarcoplasmic reticulum as the primary target of reperfusion protection. Cardiovasc. Res. 70, 170–173. 10.1016/j.cardiores.2006.03.010 16600194

[B61] Randhawa P. K. Jaggi A. S. (2014). TRPV1 and TRPV4 channels: Potential therapeutic targets for ischemic conditioning-induced cardioprotection. Eur. J. Pharmacol. 746C, 180–185. 10.1016/j.ejphar.2014.11.010 25449039

[B62] Safran M. Kaelin W. G. Jr. (2003). HIF hydroxylation and the mammalian oxygen-sensing pathway. J. Clin. Invest. 111, 779–783. 10.1172/JCI200318181 12639980 PMC153778

[B63] Schneider M. Van Geyte K. Fraisl P. Kiss J. Aragones J. Mazzone M.. (2010). Loss or silencing of the PHD1 prolyl hydroxylase protects livers of mice against ischemia/reperfusion injury. Gastroenterology 138, 1143-54.e1-2. 10.1053/j.gastro.2009.09.057 19818783

[B64] Schofield C. J. Ratcliffe P. J. (2004). Oxygen sensing by HIF hydroxylases. Nat. Rev. Mol. Cell. Biol. 5, 343–354. 10.1038/nrm1366 15122348

[B65] Semenza G. L. (2007). Life with oxygen. Science 318, 62–64. 10.1126/science.1147949 17916722

[B66] Semenza G. L. Roth P. H. Fang H. M. Wang G. L. (1994). Transcriptional regulation of genes encoding glycolytic enzymes by hypoxia-inducible factor 1. J. Biol. Chem. 269, 23757–23763. 8089148

[B81] Shahid M. Tauseef M. Sharma K. K. Fahim M. (2008). Brief femoral artery ischaemia provides protection against myocardial ischaemia-reperfusion injury in rats: the possible mechanisms. Exp. Physiol. 93, 954–968. 10.1113/expphysiol.2007.041442 18356557

[B67] Singh M. Shah T. Khosla K. Singh P. Molnar J. Khosla S.. (2012). Safety and efficacy of intracoronary adenosine administration in patients with acute myocardial infarction undergoing primary percutaneous coronary intervention: a meta-analysis of randomized controlled trials. Ther. Adv. Cardiovasc. Dis. 6, 101–114. 10.1177/1753944712446670 22562999

[B68] Takeda K. Cowan A. Fong G. H. (2007). Essential role for prolyl hydroxylase domain protein 2 in oxygen homeostasis of the adult vascular system. Circulation 116, 774–781. 10.1161/CIRCULATIONAHA.107.701516 17646578

[B69] Talan M. I. Latini R. (2013). Myocardial infarction: cardioprotection by erythropoietin. Methods Mol. Biol. 982, 265–302. 10.1007/978-1-62703-308-4_17 23456875 PMC6207951

[B70] Tello D. Balsa E. Acosta-Iborra B. Fuertes-Yebra E. Elorza A. Ordonez A.. (2011). Induction of the mitochondrial NDUFA4L2 protein by HIF-1alpha decreases oxygen consumption by inhibiting Complex I activity. Cell Metab. 14, 768–779. 10.1016/j.cmet.2011.10.008 22100406

[B71] Tsang A. Hausenloy D. J. Mocanu M. M. Yellon D. M. (2004). Postconditioning: a form of “modified reperfusion” protects the myocardium by activating the phosphatidylinositol 3-kinase-Akt pathway. Circ. Res. 95, 230–232. 10.1161/01.RES.0000138303.76488.fe 15242972

[B72] Ueda K. Takano H. Niitsuma Y. Hasegawa H. Uchiyama R. Oka T.. (2010). Sonic hedgehog is a critical mediator of erythropoietin-induced cardiac protection in mice. J. Clin. Invest. 120, 2016–2029. 10.1172/JCI39896 20484812 PMC2877931

[B73] Vavrínková H. Tutterová M. Stopka P. Divisová J. Kazdová L. Drahota Z. (2001). The effect of captopril on nitric oxide formation and on generation of radical forms of mitochondrial respiratory chain compounds in ischemic rat heart. Physiol. Res. 50, 481–489. 11702852

[B74] Verma S. K. Krishnamurthy P. Barefield D. Singh N. Gupta R. Lambers E.. (2012). Interleukin-10 treatment attenuates pressure overload-induced hypertrophic remodeling and improves heart function via signal transducers and activators of transcription 3-dependent inhibition of nuclear factor-kappaB. Circulation 126, 418–429. 10.1161/CIRCULATIONAHA.112.112185 22705886 PMC3422741

[B79] Yao Z. Tong J. Tan X. Li C. Shao Z. Kim W. C.. (1999). Role of reactive oxygen species in acetylcholine-induced preconditioning in cardiomyocytes. Am. J. Physiol. 277(6 Pt 2), H2504–H2509. 10600875 10.1152/ajpheart.1999.277.6.H2504

[B75] Zhong L. D'Urso A. Toiber D. Sebastian C. Henry R. E. Vadysirisack D. D.. (2010). The histone deacetylase Sirt6 regulates glucose homeostasis via Hif1alpha. Cell 140, 280–293. 10.1016/j.cell.2009.12.041 20141841 PMC2821045

[B76] Zhou B. Honor L. B. He H. Ma Q. Oh J. H. Butterfield C.. (2011). Adult mouse epicardium modulates myocardial injury by secreting paracrine factors. J. Clin. Invest. 121, 1894–1904. 10.1172/JCI45529 21505261 PMC3083761

[B77] Ziegler M. Elvers M. Baumer Y. Leder C. Ochmann C. Schonberger T.. (2012). The bispecific SDF1-GPVI fusion protein preserves myocardial function after transient ischemia in mice. Circulation 125, 685–696. 10.1161/CIRCULATIONAHA.111.070508 22223428

